# In vitro study of the inflammatory cells response to biodegradable Mg-based alloy extract

**DOI:** 10.1371/journal.pone.0193276

**Published:** 2018-03-14

**Authors:** Liang Jin, Jing Wu, Guangyin Yuan, Tongxin Chen

**Affiliations:** 1 Department of Allergy and Immunology, Shanghai Children’s Medical Center, Shanghai Jiao Tong University School of Medicine, Shanghai China; 2 National Engineering Research Center of Light Alloy Net Forming and State Key Laboratory of Metal Matrix Composite, Shanghai Jiao Tong University, Shanghai, China; 3 Med-X Research Institute, School of Biomedical Engineering, Shanghai Jiao Tong University, Shanghai, China; 4 Division of Immunology, Institute of Pediatric Translational Medicine, Shanghai Jiao Tong University School of Medicine, Shanghai China; University of North Texas, UNITED STATES

## Abstract

Biodegradable Mg-based alloys have shown great potential as bone fixation devices or vascular stents. As implant biomaterials, the foreign body reaction (FBR) is an important issue to be studied, where the inflammatory cells play a key role. Here, we used two inflammatory cell lines i.e. THP-1 cells and THP-1 macrophages, to evaluate the effect of Mg–Nd–Zn–Zr alloy (denoted as JDBM) extracts on cell viability, death modes, cell cycle, phagocytosis, differentiation, migration and inflammatory response. The results showed that high-concentration extract induced necrosis and complete damage of cell function. For middle-concentration extract, cell apoptosis and partially impaired cell function were observed. TNF-α expression of macrophages was up-regulated by co-culture with extract in 20% concentration, but was down-regulated in the same concentration in the presence of LPS stimulation. Interestingly, the production of TNF-α decreased when macrophages were cultured in middle and high concentration extracts independent of LPS. Cell viability was also negatively affected by magnesium ions in JDBM extracts, which was a potential factor affecting cell function. Our results provide new information about the impact of Mg alloy extracts on phenotype of immune cells and the potential mechanism, which should be taken into account prior to clinical applications.

## Introduction

Nowadays, metallic biomaterials have been widely used in clinical surgeries, e.g. bone substitute and fixative devices for total hip arthroplasty and bone fracture [1] or vascular stents and drug-eluting scaffolds for ischemic heart disease[2]. Among them, permanent metallic biomaterials, such as stainless steel and titanium alloy, have taken the absolutely major part because of their good performance in mechanical strengths and biocompatibility[3]. However, the drawbacks including second surgery, chronic inflammation and in-stent restenosis have been gradually recognized during their clinical use [4, 5].

Recently, Magnesium-based biomaterials have been a research hotspot as biodegradable implant devices due to their good mechanical properties [6] and biodegradability [7]. The intermediate degradation products including magnesium hydroxide (Mg(OH)_2_) and hydrogen gas could be completely absorbed in human body or engulfed by macrophages [8, 9]. However, the excessive biocorrosion rates of magnesium alloy raised concern about the roles Mg alloy might play in pathophysiology and toxicology at the accumulative location of body. In addition, although magnesium has been used in various clinical purposes such as cerebral palsy prevention[10], high dose magnesium might induce hypermagnesaemia [11]. Thus, it is necessary to evaluate biological influence of Mg-based alloy, especially in monocytes and macrophages.

Monocytes and macrophages play a pivotal role in FBR triggered by implantation of biomaterials [12]. In brief, macrophages, differentiated from recruited monocytes, are assembled at the surface of implants to ingest foreign material and recruit other cells or fuse into foreign body giant cells to participate in wound healing process [13]. Meanwhile, macrophages can be polarized into pro-inflammatory subtype (M1) expressing IL-6,TNF-α or anti-inflammatory subtypes (M2a,b,c) secreting IL-10,TGF-β, once recruited to the place around the implant [14]. Not limited to common characteristics of FBR, Mg-based materials have some special effects due to their biodegradable characteristics. For instances, magnesium corrosion products could exert anti-osteoclasts activity by inhibiting nuclear factor-κB (NF-κB) activation [15]. In addition, macrophages may inversely interfere with the degradation process of Mg alloy through phagocytosis of second phase [16][17]. Currently, little is known about the influence of Mg-based alloy on immune cells. In present study, we tested the physiochemical property of an Mg-based alloy (Mg–2.1Nd–0.2Zn–0.5Zr, wt %, abbreviated as JDBM) which was developed for cardiovascular stents, as well as its biological effects on monocytes and macrophages, in order to provide new insight into the clinical translation for this alloy. THP-1 human monocytic cell line and its derived macrophages were used [18] because of their high similarity with primary monocytes and macrophages in biological function [19].

## Methods and materials

### Magnesium alloy samples and extract preparation

The detailed composition and ingot of JDBM used in this study have been described in previous studies [20,21]. Disc samples for the experiments with a diameter of 18 mm and a height of 2.0 mm were ultrasonic cleaned with ethanol and acetone for 10 minute and then were sterilized by exposing under ultraviolet for 1h before used. Extracts were prepared according to ISO-10993 guideline. In brief, Disc samples were immersed in cell culture medium, RPMI 1640 (Gibco TM, Invitrogen), with the surface area1/volume ratio of 1.25 cm^2^/ml for 72h (5% CO2 at 37°C). After that, extracts were harvested, filtered by 0.2μm filter and stored at 4°C. To detect a dose-dependent effects, the extracts were diluted with RPMI 1640 into concentrations of high (100%), middle (50%) and low (10% or 20%), respectively. The magnesium ion concentrations, pH value and osmotic pressure of the extracts were measured by inductively coupled plasma atomic emission spectrometer (ICP-AES, Perkin-Elmer Optima 2000, USA), pH detector (PB-10, Sartorius, Germany) and Freezing point osmometer (Osmomat 3000,USA) (Table 1), respectively.

**Table 1:** The physicochemical characteristics of JDBM extract.

Samples	pH	Osmolality(mOsm/kg)	Mg^2+^ concentration(mg/L)
Ctr(1640 Medium)	7.5±0.17	283.2±1.5	10
10% Extract	7.53±0.2	295.7±2.4	98.29±42.6*
20% Extract	7.86±0.14	307.2±4.3*	184±41.76*
50% Extract	8.02±0.11*	338.7±14.2*	531±167.3*
100% Extract	8.24±0.12*	390.7±12.1*	1113.2±171*

*P < 0.05 VS Ctr.

### Cell culture and differentiation

The THP-1 cell line was obtained from culture collection of the Chinese Academy of Sciences, Shanghai, China and kept at 1×10^^6^/ml in RPMI 1640 medium supplemented with 10% heat- inactivated fetal bovine serum(FBS) and 1% penicillin-streptomycin (PS). THP-1 cells were treated with 50ng/ml Phorbol 12-myristate 13-acetate (PMA, Sigma-Aldrich, USA) for 48h to differentiate into macrophages. Both THP-1 cells and macrophages were seeded in 24- well cell culture plate (Coring, USA) at 2×10^^5^/ml or 96- well cell plate at 5×10^^4^/ml with JDBM extracts containing 10% FBS and 1% PS for 24h or 72h (5% CO2 at 37°C). RPMI 1640 medium alone was set as control group.

### Cytotoxicity and cytomorphology test

For assessing the impact of Mg alloy extracts on cytotoxicity, THP-1 cells or macrophages were seeded into 96-well along with extracts diluted into different concentration mentioned above for 24h and 72h. Then, 100μl RPMI 1640 containing CCK-8 solution (Dojindo, Kumamoto, JAPAN) was added to the plate and the supernatant was collected by centrifugation. Finally, plates were re-incubated for 3h (5% CO2, 37°C) followed by reading on a microplate reader (BioTek, Winooski, VT, USA) at 450 nm test wavelength. The cell relative growth rate (RGR) was calculated as following: RGR = OD _test_ /OD _control_ × 100%. To validate our results, cell viability was also measured by lactate dehydrogenase (LDH) cytotoxicity assay kit (Beyotime, China) according to the protocol. THP-1 cells or macrophages were seeded into 24-well with different concentration extracts for 72h. The cellular morphology was captured by light microscope at initial or ultimate time.

### Flow cytometry analysis

#### Cell cycle

After co-culture with the extracts, THP-1 cells were collected, washed once with cold phosphate buffered saline (PBS), fixed in ice-cold 70% ethanol and stored at -20°C for overnight. Then, cells were washed again with PBS and suspended in 500μl PBS containing 100μg/ml RNase A and 150μg/ml propidium iodide (PI, Beyotime, China). Finally, after 4°C incubation for 30min, cells were collected and subjected for flow cytometry (FACS, Canto II, BD, USA).

#### Cell apoptosis and necrosis

Apoptotic kit (BD, USA) including annexin V-FITC and PI were used for analyzing the effect of JDBM extracts on apoptosis and necrosis of THP-1 and macrophages, respectively. Briefly, treated cells were harvested, washed, and suspended in Annexin-binding Buffer. After staining with Annexin-V-FITC and PI for 15min in the dark, the apoptosis or necrosis rate was measured by FACS.

#### Cell phagocytosis

For the evaluation of phagocytosis, macrophages were treated with the JDBM extracts for 24h and then were added with Carboxylate-modified red fluorescent latex beads (10ul/ml medium) with a mean diameter of 2μm (L3030, sigma-Aldrich, USA) for another 4h. After twice washing with PBS, cells were collected and measured by FACS.

#### Cell differentiation

During the differentiation inducement by PMA for THP-1 cells, both CD14 and CD54 molecules were demonstrated to be up-regulated and TLR-2 to be down-regulated according to previous reports [18,22]. Therefore, these markers in cell surface were used for FACS analysis in this study to supervise the effect of extracts on the differentiated status of THP-1, with detailed experimental process similar to the aforementioned. All data for FACS analysis were analyzed using Flowjo 7.6.

### Real-time quantitative PCR (RT-qPCR) analysis

The mRNA expression level of apoptotic and cell cyclic related genes was assayed by RT-qPCR, with *GAPDH* used as the housekeeping gene for normalizing. The THP-1 cells were seeded into 24-well cell plate along with extracts in different concentration for 24h. Total RNA was isolated using TRIZOL reagent (Invitrogen, USA) according to standard protocol. The cDNA synthesis was performed according to the protocol of manufacture (TOYOBO, Japan). Bio-Rad C1000 was finally used for RT-qPCR analysis with SYBR green mixes (TOYOBO, Japan). The expression levels of target genes were evaluated using ΔΔCt method and were normalized to *GAPDH*. The primer sequences of the genes were list in S1 Table.

### Inflammatory cytokine test

Macrophages were treated with or without 200ng/ml LPS (sigma, USA) in combination with JDBM extracts. Supernatants were collected after 24h and stored at -80°C until measurement. TNF-α level was tested by ELISA (DAKEWE, China) according to manufactures’ instructions. The minimal concentration for TNF-α was 30pg/ml.

### Cell scratching test

Macrophages were seeded into 6-well plate at 5×10^5^ cells per well. After 24h, adhered macrophages were scratched with pipette tips, washed with PBS and added with various extracts or PRMI 1640. Images were captured by light microscope at 0h, 24h and 48h. The migration ability of macrophages was quantified by subtracting the scratch distance at 0h from the area at 24h and 48h, respectively, with NIH ImageJ 1.46 software.

### Statistical analysis

All experiments were repeated three times independently and relevant data were analyzed using SPSS software (Version 19, IBM Co. USA). Differences among groups were analyzed by one-way ANOVA with Turkey HSD or Dunnett T3 methods. All results were interpreted as means ± standard deviation (SD). Two-tailed *P <* 0.05 was considered as statistical significance.

## Results

### The cytotoxic effect of JDBM extracts on THP-1 cells and macrophages

**Fig 1:**
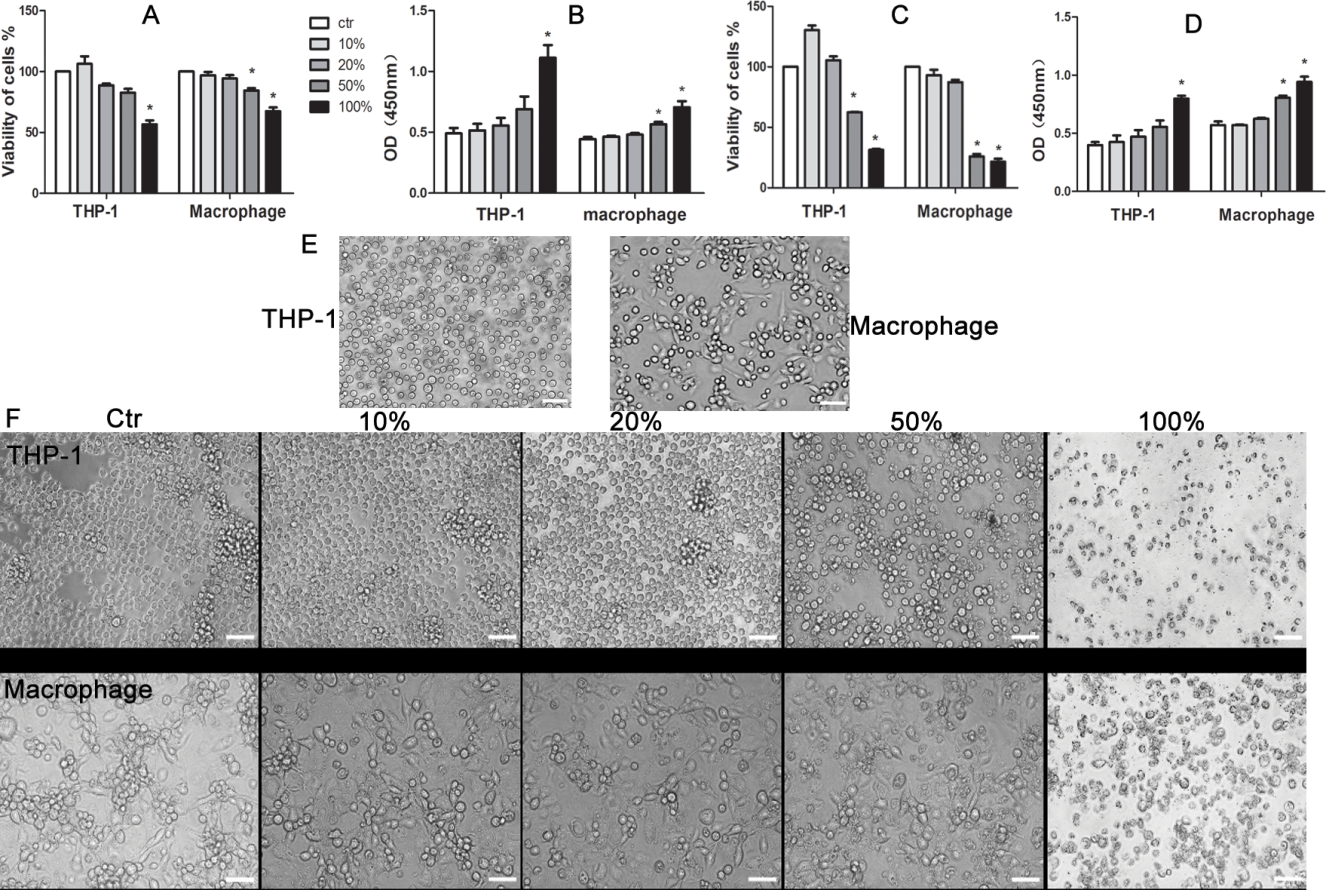
The effect of JDBM extracts cell viability of THP-1 cells and macrophages. CCK-8 test (A andC) and LDH tests (B andD) were assayed at 24h (A andB) and 72h (C andD). (E and F) Cytomorphology change of THP-1 cells and macrophages in the presence of JDBM extracts was observed at 0h and 72h. *P < 0.05 vs ctr. Abbrevations: ctr, control which was culture in RPMI 1640 medium. Scale bar = 100μm.

No cytotoxicity was detected for THP-1 cells and macrophages in the presence of extracts with a concentration of 10% and 20% for 24h or 72h (Fig 1A and 1C). A significant reduction in cell viability induced by 50% extracts after the treatment for 24h was observed in macrophages, while the viability of THP-1cells were likely unaffected until 72h treatment. Of note, the extracts adversely affected cell viability of both THP-1 cells and macrophages in a time-dependent manner (Fig 1B and 1D). A similar result was also observed in LDH release assay, indicating the strength of the results. In addition, there was no difference on cellular morphology of THP-1 cells and macrophages between low concentration groups (10% and 20%) and control group after incubation for 72h. However, the number of THP-1 cell was decreased in the presence of 50% and 100% extract and in a concentration-dependent manner. Furthermore, The macophages were shown round type, smaller size and less pseudopodia (macrophage) in the presence of 50% and 100% extract compared with that in the rest groups (Fig 1E and 1F).

### Apoptosis of THP-1 cells and macrophages induced by JDBM extracts

**Fig 2:**
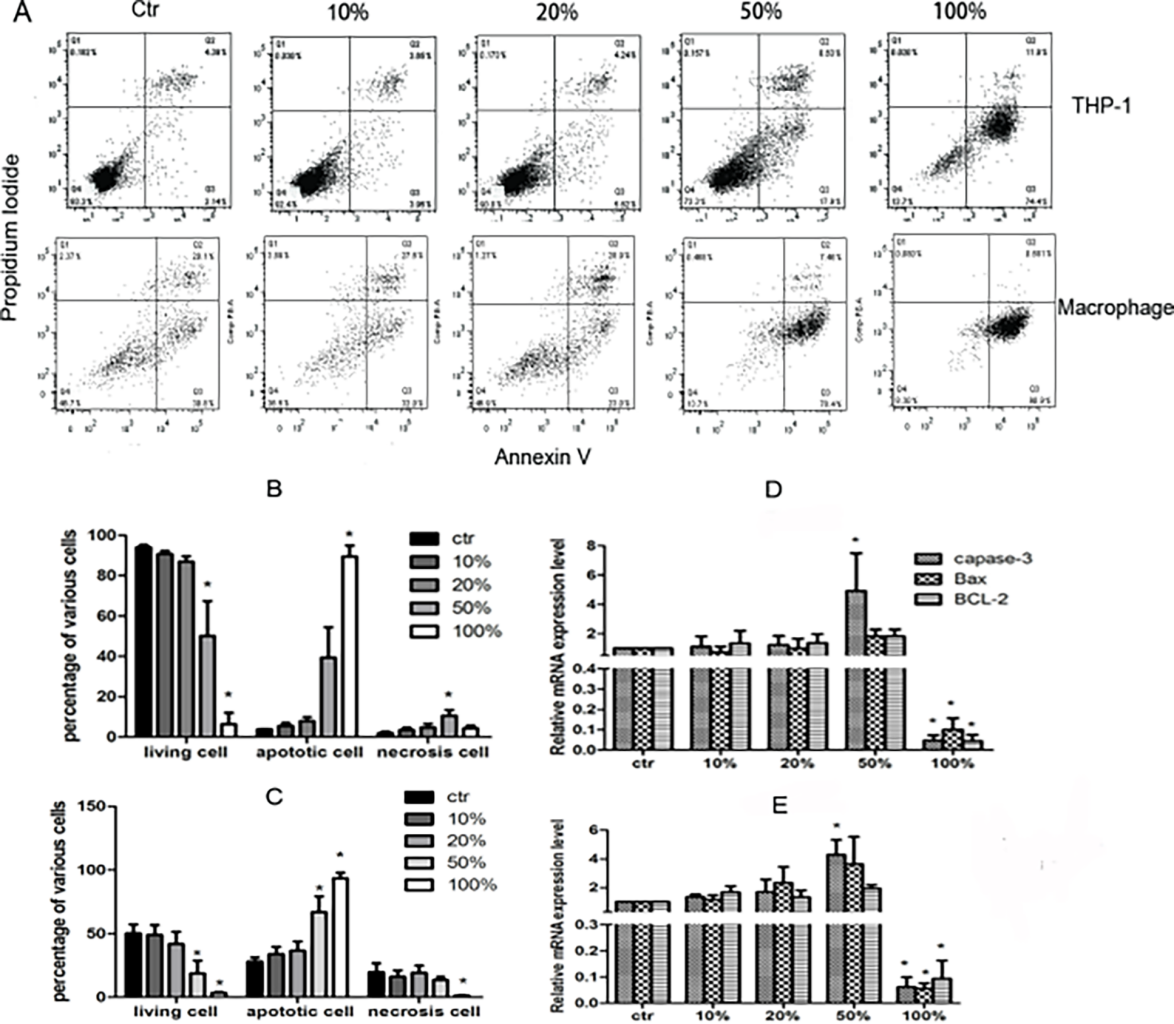
The test on death modes of THP-1 cells and macrophages induced by JDBM extract with different concentration or RPMI 1640 medium (Ctr). (A) A representative result of cell performance in various extract assayed by FACS for 72h. (B and C) Percentage of THP-1 cells and macrophages in different death mode, respectively, which cultured in JDBM extract or ctr for 72h.(D and E) The expression of aptosis-related genes in THP-1 cells and macrophages treated with indicated *P < 0.05 vs ctr.

Compared with control group, both THP-1 cells and macrophages had no significant difference in apoptosis and necrosis, when they were incubated with 10% or 20% extracts for 72h (Fig 2A). On the contrary, either 50% or 100% extract significantly induced apoptosis and necrosis in the THP-1 cells and macrophages (Fig 2B and 2C). The *capase-3* expression in THP-1 cells and macrophages in the presence of the 50% extract was higher than that in the control group, whereas the expression of *BCL-2* and *Bax* had no significant difference. When the concentration of extracts was increased to 100%, expression of all the genes were significantly decreased in comparison with control group (Fig 2D and 2E).

### The effect of JDBM extracts on the cell cycle analysis of THP-1 cells

**Fig 3:**
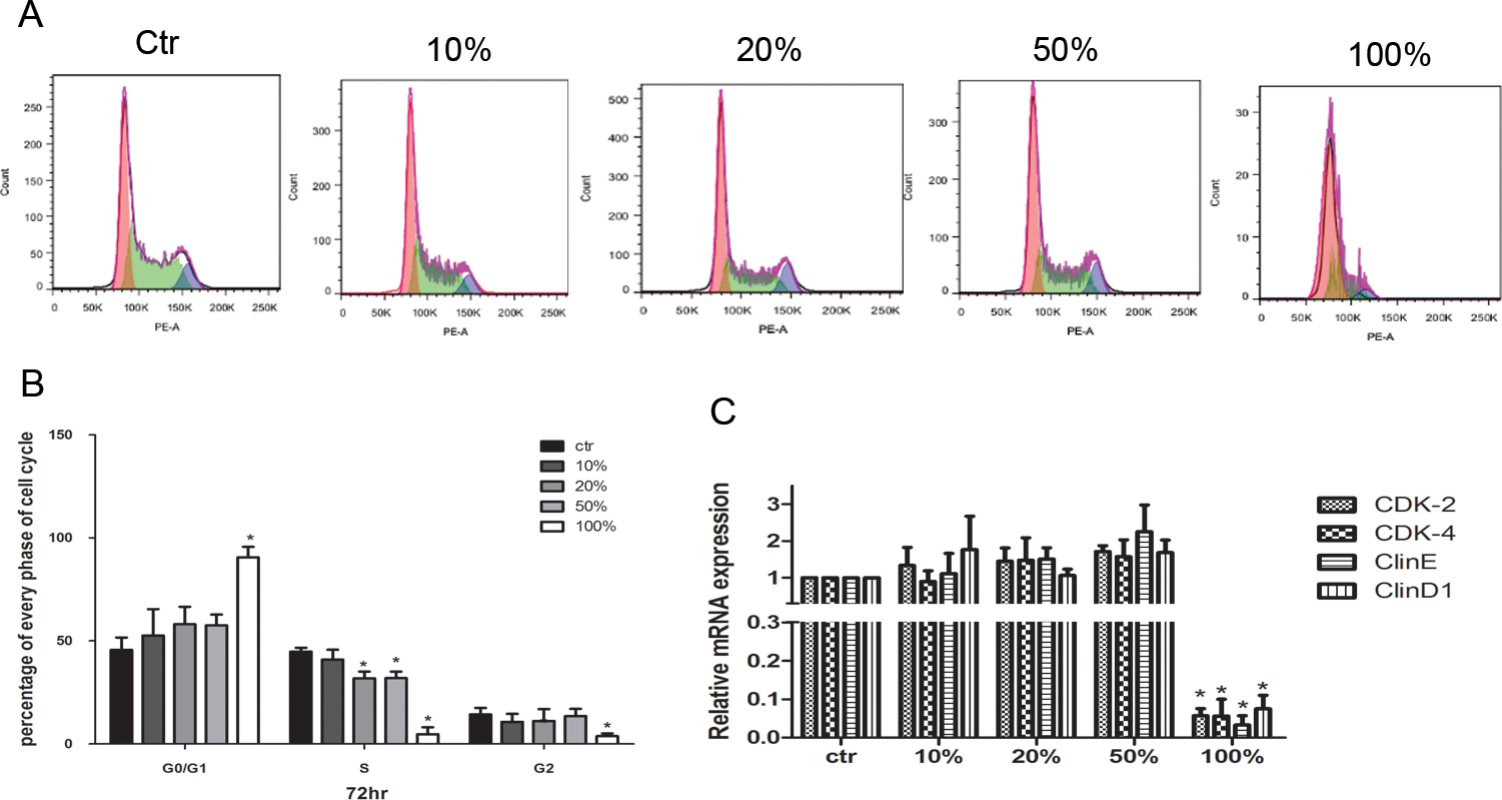
The impact JDBM extract on cell cycle of THP-1 cells. (A) A representative result of cell cycle after treatment of RPMI 1640 medium (Ctr) or JDBM extract for 72h. (B) Statistical results of cell cycle of THP-1 cells in extract culture for 72h. (C) The expression of cell cycle related genes of THP-1 cell treated with indicated for 24h *P < 0.05 vs ctr.

After 72h treatment for THP-1 cells, the cell cycle was assessed by FACS with three index: G0/G1(red), S(green) and G2(blue) (Fig 3A). We observed a similar performance in cell cycle among the groups of 10%, 20% and 50% extracts, although the ratio of S phase cells was slightly decreased only by the treatment with 20% and 50% extract in comparison with control group (*P* <0.05). Of note, compared with the control group, a prominent decrease in the ratio of S and G2 phase cells and an increase in the ratio of G0/G1 phase cells were observed in presence of 100% extract (Fig 3B, *P* <0.05). The mRNA expression levels of *cyclin D1*, *cyclin E*, *CDK2* and *CDK4* [23] of THP-1 cells, were decreased significantly by the treatment of 100% extract in comparison with the control group (*P*< 0.05 for all, Fig 3C), but no difference was observed between other extracts treated groups with a concentration less than 100% and the control group.

### The impact of JDBM treatment on the differentiation of THP-1 cells

**Fig 4:**
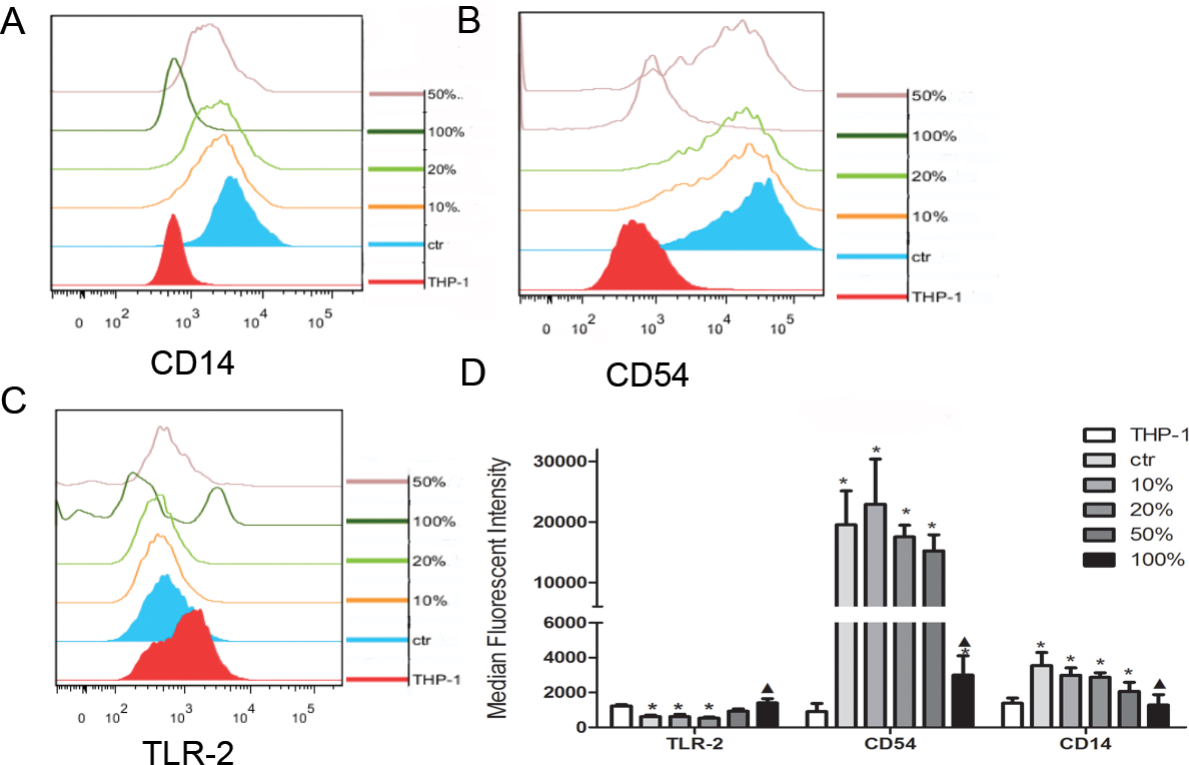
The impact of PMA and JDBM extracts on cell differentiation of THP-1 cells into macrophage. PMA treatment alone was pos-ctr, PRMI 1640 treatment alone was neg-ctr. (A-C) representative results of FACS test on CD14, CD54 and TLR-2, respectively, after induced by JDBM extract cell differentiation performance in various extract assayed. THP-1 cell without PMA was THP-1 and with PMA was ctr. (D) Statistical results of the expression change on CD14, CD54 and TLR-2 of cell under various extracts. *P < 0.05 vs ctr, ^▲^*P* < 0.05 vs THP-1.

To assess whether extract might modulate the THP-1 differentiation, we inspected the expression of *CD14*, *TLR-2* and *CD54* of THP-1 cells. In consistence with the previous reports [18,22], TLR-2 was down-regulated, while both CD54 and CD14 were up-regulated, after a stimulation of PMA for THP-1cells (Fig 4D) in comparison of cells without PMA treatment. Furthermore, we observed that the differentiated status of cells treated with 10% and 20% extract in combination with PMA was similar to that of cells treated with alone PMA, indicating that low concentration of extract might have no effects on THP-1 cell differentiation. However, we still did not observe any effects on promoting the differentiation in presence of PMA treatment if cells were simultaneously treated with 100% extracts (Fig 4D). Interestingly, there was a significant increase in the *CD14* and *CD54* expression in cells treated with the combination of 50% extracts and PMA compared with cells treated with PMA alone (Fig 4A and 4B), but no significant difference was found for the expression of *TLR-2* (Fig 4C).

### The impact of JDBM extracts on the phagocytic function and migration of macrophages

**Fig 5:**
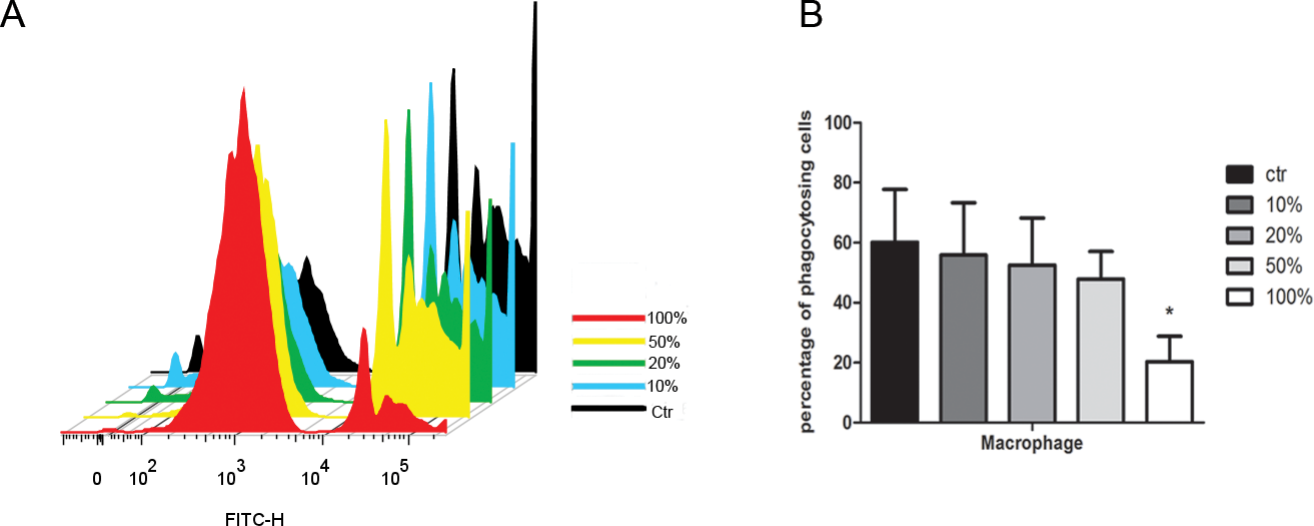
The impact of JDBM extracts on the phagocytic function of macrophages. (A) A representative result of cell phagocytic performance with beads for 4h after induced by JDBM extracts or RPMI 1640 (ctr) for 24h by FACS. (B) Statistical results of FACS. *P < 0.05 vs ctr.

**Fig 6:**
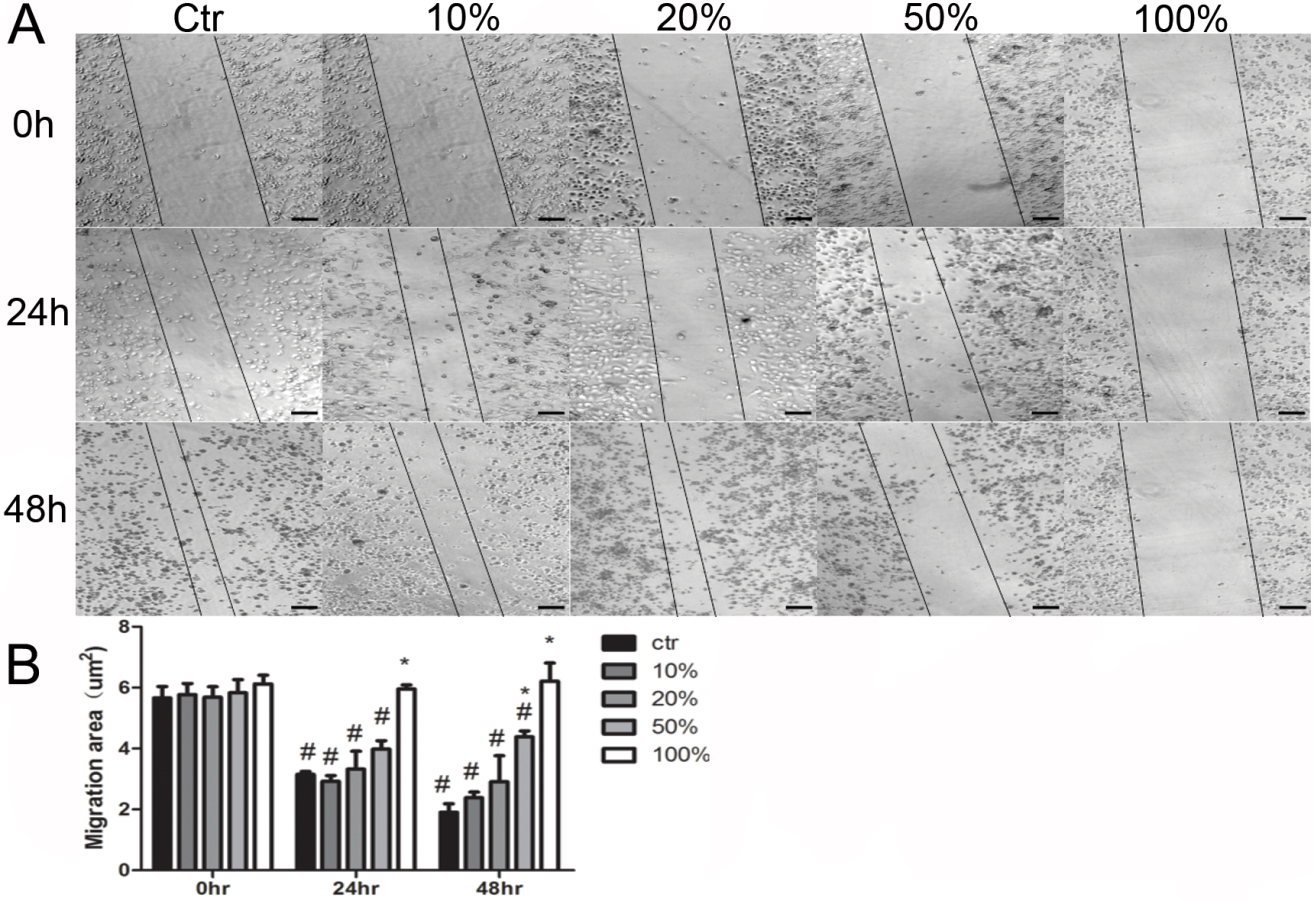
The impact of JDBM extracts on the migration function of macrophages. (A) A representative result of scratch assay after treated with JDBM extracts or RPMI 1640 (ctr) at 0, 24, 48h, respectively. Scale bar = 100μm. (B) statistical results of scratch assay. ^#^*P* < 0.05 vs 0hr related group*P < 0.05 vs 24hr related group.

To explore whether the phagocytic activity of macrophages could be influenced by JDBM extracts, fluorescent latex beads were added into macrophages in combination with JDBM extracts of different concentration. Compared with the control group, there was no significant difference in phagocytic ability (*P*>0.05 for all), except for the group of treatment with 100% extract in which a significant inhibition of phagocytic function was observed (Fig 5A and 5B, *P*< 0.05). As shown in Fig 6A, similarity, JDBM extracts negatively affecting the migration of cells was observed only under the treatment with 100% extract at 24h. Furthermore, the cell migration ability was partly and totally impaired by a 50% and 100% extract treatment at 48h, respectively (Fig 6B).

### JDBM extracts modulated the inflammatory responses of macrophages

**Fig 7:**
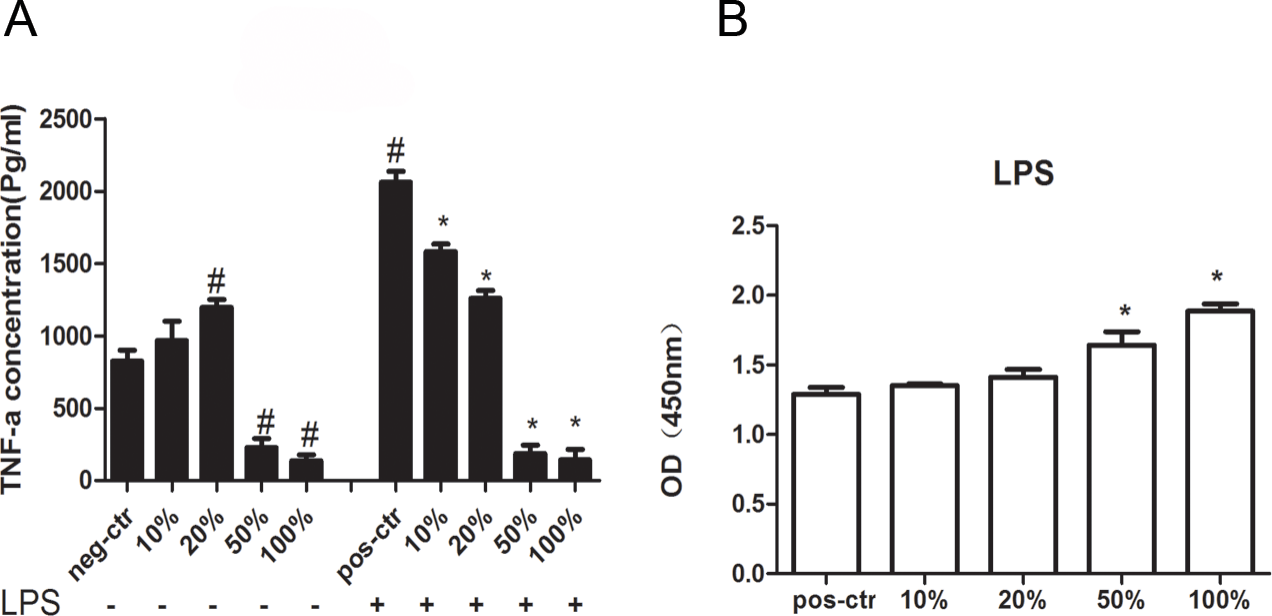
JDBM extracts regulated the inflammatory responses of macrophages. (A) TNF-α expression level in cell treated with different extract concentration with or without LPS (200ng/ml) for 24h by ELISA.RPMI 1640 treatment alone was neg-ctr, RPMI 1640 with LPS treatment was pos-ctr. (B) LDH test for assessment of cell viability of macrophages treated with indicated. ^#^*P* < 0.05 vs neg-ctr, *P < 0.05 vs pos-ctr.

To further explore the inflammatory response to JDBM extract for macrophages, we assessed the *TNF-α* expression in macrophages. As shown in Fig 7A (left), compared with the group without any treatment, the *TNF-α* level in macrophages had no significant difference when treated with 10% extract but was increased by treatment of 20% JDBM extracts. Furthermore, as shown in Fig 7A (right), TNF-α expression in LPS stimulated macrophages was higher than in non-stimulated group. Interestingly, TNF-α expression of both groups treated with 10% and 20% extracts in the presence of LPS were lower than that of group treated with LPS alone, indicating that the inflammatory response to JDBM extracts was at least partly dependent on LPS stimulation. Of note, we observed that the inflammatory response to JDBM extracts with medium and high concentration was independent of LPS, given the observation that the TNF-α expression was decreased by both a concentration of 50% and 100% JDBM extracts, no matter whether the LPS was added to culture medium. For the further study of relationship between cytotoxicity and inflammation, we also tested the LDH release in all groups with LPS treatment. As shown in Fig 7B, only the addition of JDBM extracts with 50% and 100%, not the low concentration (10% and 20%), induced the LDH release. Because LDH release is a symbol of cell death, the increased immune cell death was the potential mechanism underlying the observation that TNF-a expression was inhibited by 50% and 100% JDBM extracts.

### The cytotoxicity impact of MgCl_2_ or alkaline cell culture medium on THP-1 cells and macrophages

As the degradation of JDBM might result in the release of Mg ions and the rise of pH and osmolality in the medium, it was necessary to reveal the effects of the degradation on cell viability. We used RPMI 1640 containing MgCl_2_ (1g/L, 400mOsm/kg) or RPMI 1640 medium with a pH of 8.5 adjusted by NaOH as culture mediums to mimic 100% JDBM extracts (Table 1). After THP-1 and macrophages were respectively cultured with above mediums for 72h, cytotoxicity was detected by Annexin V/ PI using FACS. RPMI 1640 medium with a high pH value of 8.5 was unable to impair the cell viability in comparison with the cells treated with normal RPMI 1640 medium. However, the impaired cell viability was observed in both THP-1 cells and macrophages if the Mgcl_2_ was added to the RPMI 1640 medium (S1 Fig), and this adverse influence on cytotoxicity was similar to that in the treatment with 100% JDBM extract (Fig 2A).

## Discussion

Mg-based alloy has been widely used in the clinical management. Nevertheless, FBR is an obstacle for its clinical application. As indicated by previous reports, biomaterials might influence the inflammation response, phagocytosis, migration of macrophages and monocytes [12,24]. Furthermore, Mg-based alloy could degrade according to the following chemical equations: Mg+H_2_O—Mg(OH)_2_+H_2_ and Mg(OH)_2_+2Cl^-^—MgCl_2_+2OH^-^[25]. Therefore, it should be taken into consideration that massive accumulation of these degradation products due to overburdening the organism regulation or burst release would in turn deteriorate local physical condition [26]. In the present study, we found JDBM extracts in a middle concentration (50%) promoted apoptosis and partly impaired the cell viability of macrophages. For the treatment of JDBM extracts with high concentration (100%), the cells hardly survived even in a short time, while the low concentration (10% and 20%) of JDBM extracts had no effects on cell viability. According to current ISO standard of Part 5, biomaterials is considered as nontoxic if cell viability sustained higher than 75% [27]. We found only the extracts with low concentration could reach the non-toxic standard. However, THP-1 was more adaptive than macrophages to 50% extract as indicated by results of CCK-8 and LDH test. The non-adherent and high proliferative property for THP-1 cells may account for this observation.

Apoptosis was considered as protective effects through removing unwanted cell to maintain homeostasis, while necrosis was normally harmful to bodies [28,29]. Furthermore, Capase-3 protein was deemed as an executor and was involved in terminal process of apoptosis [30], while necrosis might induce broken of membrane integrity and uncontrolled release of intracellular contents into extracellular [31]. Our results showed middle concentration extract of JDBM promoted cell apoptosis, while high concentration led to necrosis. However, whether other non-classical death pathways may participate in cell response to Mg-based alloy would require further investigations.

It was controversial that whether Mg-based alloy could affect cell cycle according to previous studies. For instance, there was a cell cycle arrest in the presence of low concentration of Mg-alloy based on the observation of a slightly reduced percentage of both S and G0/G1 phase cells [32]. Additionally, Mg-alloy treatment might induce an increased concentration of extracellular magnesium, leading to mild stimulation of cell proliferation [33]. However, the growth of human bone marrow mesenchymal stem cell (hBMSCs) was inhibited with treatment of Mg-alloy extract [34]. In addition, high but not the low concentration of Mg-6Zn extract could inhibit the proliferation of both intestinal epithelial (IEC-6) and bile duct epithelial cells [35,36]. In consistence with these findings, we observed a dose-dependent effect of JDBM extracts on the inhibition of THP-1 proliferation.

During the early process of FBR, monocytes were mobilized to the biomaterials through adhesion factors and further differentiated into macrophages [37]. However, little was known about what kind of role of degradation production of Mg-alloy might play in the cell differentiation from monocytes to macrophages. According to our results, treatment with high concentration JDBM extracts might modulate the differentiated status, which was not affected, however, in the presence of low and middle concentration. This result might be important for future application of JDBM extracts.

As demonstrated, there were two types of macrophages: the first one was the tissue-resident macrophages [38] and the second one was differentiated from monocytes which were migrated to biomaterial by chemokines such as MCP-1 or uPAR[39, 40]. Both of them have reliable biological function and phenotypic plasticity [8,41]. We found an impaired migration ability of macrophage only in the presence of high concentration extract but not in low or middle concentration extract. Therefore, our results might have implications on the involvement of Mg-alloy in the process of FBR.

Macrophages could regulate the degradation of biomaterials by secreting proteolytic enzymes, matrix metalloproteinases and phagocytosis [42,43]. Macrophages maintained the ability of clearing intracellular mycobacterium segments via phagocytosis under an environment containing magnesium particles [44]. Our results further revealed the phagocytosis of macrophage could be only affected by high concentration extract of Mg-based alloy. However, the impacts of JDBM extracts on the expression of phagocytosis related receptors such as class A scavenger receptor or mannose receptor [42], was not fully understood, and therefore needs further investigation.

It was reported that Magnesium inhibited inflammatory response through down-regulation of TLR4/NF-kB, activation of phosphoinositide 3-kinase (PI3K)/Akt pathway or the inhibition of HMGB1 secretion [39, 45, 46]. Although a low concentration of 20% JDBM extracts slightly promote the TNF-a expression due to the early FBR process as previously indicated [37], we confirmed the inhibition of TNF-a expression if cells were treated with low concentration of extract under the stimulation of the LPS. Interestingly, the inhibition of inflammatory response was revealed to be independent of LPS when immune cells were treated with JDBM extracts with 50% or 100% concentration, which might be attributed to the adverse effect of magnesium concentration on cell viability.

Obviously, the function of immune cell was dependent on cell viability, while the magnesium concentration of Mg-alloy extract is critical to affect cell viability. Hence, it was important to clarify which component of extracts contributed to decreased cell viability. Previous reports found that the medium transiently adjusted to pH 8.0–9.0 through adding NaOH for 24h had no influence on viability of Human endothelial vein cells [47]. In consistence with this report, we also observed an unaffected cell viability in the presence of a relatively high pH value adjusted by NaHCO_3_/5%CO_2_ buffer system. However, the viability of L929, BMSCs, MC3T3-E1 and osteoblast were reportedly to be damaged by indicated MgCl_2_ medium and the extracts of high-pure Mg or alloy (1g/L) [48, 49], which was also in accordance with our observations that the viability of THP-1 cells and macrophage was impaired by MgCl_2_ and JDBM extracts. According to our results, no less than 5 times dilution of JDBM extract was recommended to be adopted for in vitro function research of monocyte and macrophages cells to avoid impairing cell viability.

This research had several limitations. Firstly, although magnesium ion played a critical role in immune cell function, the effect of other ions of JDBM might have been missed. Secondly, using primary cell may be better than cell line to interpret the results in the experiments. Finally, in vivo study is required to validate the in vitro results.

## Conclusion

JDBM extracts with middle or high concentration might modulate the viability, apoptosis and necrosis of immune cells, while low concentration extract seemed to have no influence. The viability of the immune cells was inhibited by Mg ions produced by degradation of JDBM. JDBM extracts might reduce inflammatory response of macrophages stimulated by LPS through magnesium-based adverse effect on immune cell. Our results provided new evidences on the influence of Mg based alloy on monocyte and macrophages, in terms of phenotype and the potential mechanisms.

## Supporting information

S1 Fig Cytoxicity THP-1 (up) and macrophages (down) induced by MgCl_2_ or alkaine cell culture medium. Annexin V/PI were assayed by FACS after culture in pH8.5, 1g/L ml MgCl_2_ and RPMI 1640 (control) for 72h.
(TIF)

S1 Table Primers used for real-time PCR. (DOCX)
