# Supplementary figures and images for: In vitro study of the inflammatory cells response to biodegradable Mg-based alloy extract
Source: PLoS One. 2018 Mar 14;13(3):e0193276. doi: 10.1371/journal.pone.0193276 (PMC5851599; doi:10.1371/journal.pone.0193276)

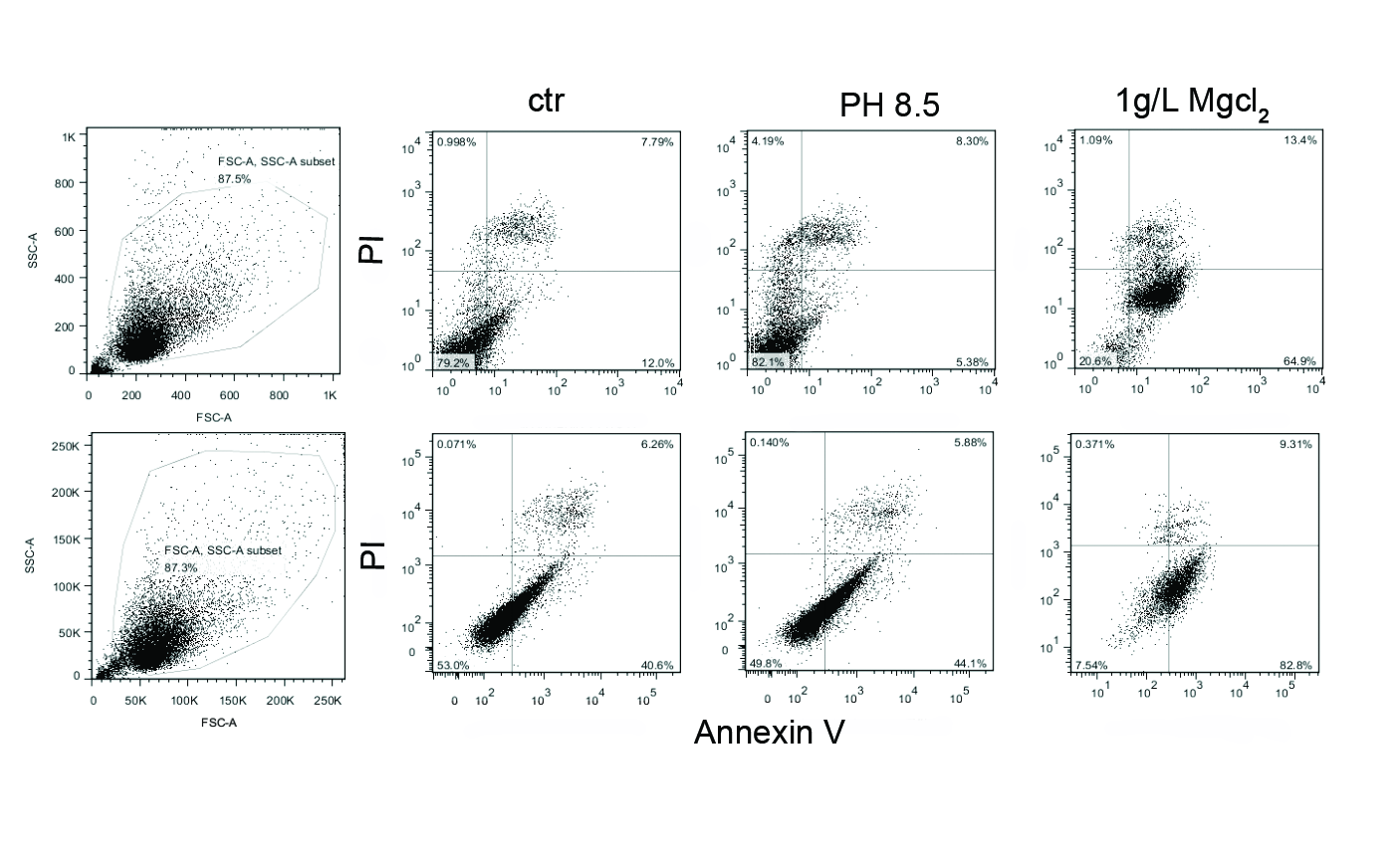

Supplement: S1 Fig — Annexin V/PI were assayed by FACS after culture in pH8.5, 1g/L ml MgCl2 and RPMI 1640 (control) for 72h. (TIF) [file pone.0193276.s001.tif]
