# Supplementary material for: In vitro study of the inflammatory cells response to biodegradable Mg-based alloy extract
Source: PLoS One. 2018 Mar 14;13(3):e0193276. doi: 10.1371/journal.pone.0193276 (PMC5851599; doi:10.1371/journal.pone.0193276)
Supplement: S1 Table — (DOCX) [file pone.0193276.s002.docx]

Supplementary table 1. Primers used for real-time PCR

| Genes | Forward (5’-3’) | Reverse(5’-3’) |
| --- | --- | --- |
| Bax | TGCTTCAGGGTTTCATCCAG | GGCGGCAATCATCCTCTG |
| Bcl-2 | GGCTGGGATGCCTTTGTG | CAGCCAGGAGAAATCAAACAGA |
| Capase-3 | GCAGCAAACCTCAGGGAAAC | TGTCGGCATACTGTTTCAGCA |
| Cyclin D1 | TCTACACCGACAACTCCATCC | GTGTTTGCGGATGATCTGTTT |
| Cdk 4 | ATGTTGTCCGGCTGATGGA | CACCAGCGTTACCTTGATCTCCC |
| Cdk 2 | TCCAGGATGTGACCAAGCC | CTGAGTCCAAATAGCCCAAGG |
| Cyclin E | AGTGGCGTTTAAGTCCCCTG | ATACAAGGCCGAAGCAGCAA |
| GAPDH | GGAGAAGGCTGGGGCTCAT | TGATGGCATGGACTGTGGTC |
